# *In Vitro* Model of Stretch-Induced Lung Injury to Study Different Lung Ventilation Regimens and the Role of Sedatives

**Published:** 2020-05-15

**Authors:** Yusuke Mitsui, Sophia Koutsogiannaki, Miho Fujiogi, Koichi Yuki

**Affiliations:** 1 Department of Anesthesiology, Critical Care and Pain Medicine, Cardiac Anesthesia Division, Boston Children’s Hospital, Boston, Massachusetts, 02115, USA; 2 Department of Anaesthesia, Harvard Medical School, Boston, Massachusetts, 02115, USA

**Keywords:** Cyclic stretch, mechanical ventilation, cell death, sedatives

## Abstract

**Background:**

Currently lung injury is managed conservatively through supportive care including mechanical ventilation. However, mechanical ventilation can also cause additional lung injury due to over-stretch along with atelectasis and cytokine release. Here we developed an in vitro mechanical ventilation model using cyclic stretch of lung epithelial cells to mimic high and low tidal volume (TV) ventilation strategy, so that we could use this platform for pathophysiology analysis and screening for therapeutic drugs.

**Method:**

We subjected MLE-15 cells to the following treatments. 1) No treatment, 2) lipopolysaccharide (100 ng/mL) stimulation for 24 hours, 3) mechanical stretch initiated at 6-hour time point for 18 hours, 4) LPS stimulation at time point 0 hour, and mechanical stretch was added at 6-hour time point for 18 hours. Biaxial cyclic stretch with a triangular wave was given via the Flexcell FX-6000 tension system to mimic low and high TV. Anesthetics dexmedetomidine and propofol were also tested.

**Result:**

Our high TV mimic stretch increased cell death, while low TV mimic stretch did not affect the degree of cell death. Using this system, we examined the effect of sedatives commonly used in intensive care units on cell death and found that dexmedetomidine attenuated necrosis associated with stretch.

**Conclusion:**

We described the in vitro cyclic stretch system mimicking high and low TV ventilation. High TV mimetic was associated with increased cell death. Dexmedetomidine attenuated the degree of cell death.

## Introduction

Although ventilatory support is often required in patients with pulmonary dysfunction to maintain adequate gas exchange, many clinical studies have suggested that mechanical ventilation by itself can induce lung injury (ventilator-induced lung injury; VILI) as a result of over-stretch (volutrauma), atelectasis (atelectrauma) and pro-inflammatory cytokine production (biotrauma) [1]. Alveolar epithelial cells are major cell types in the alveoli and play a major role in gas exchange. Accordingly, cyclic stretch has been applied to alveolar epithelial cells to study the pathophysiology of VILI derived from overdistension-induced injury. It has been shown that cyclic stretch facilitated cell death as the pathophysiology of volutrauma [2].

When setting up a mechanical ventilation, tidal volume (TV) and positive end-expiratory pressure (PEEP) are important parameters. The landmark study by the Acute Respiratory Distress Syndrome Network demonstrated that mechanical ventilation with low TV (6 mL/kg) strategy (“lung protective strategy”) was associated with a lower mortality and increased days without ventilator use than mechanical ventilation with “high (traditional)” TV (12 mL/kg) strategy [3]. PEEP is set high in the lung protective strategy compared to the traditional TV strategy to attenuate the decruitment of the lung and improve oxygenation.

The purpose of this study is to mimic low and high TV mechanical ventilation using the cyclic stretch system. Because mechanical ventilation is typically instituted for patients with pulmonary dysfunction, we also mimicked the scenario that patients with the primary lung injury would undergo mechanical ventilation. Because there is no specific therapy against VILI, the availability of an *in vitro* system would be important to examine potential therapeutics. During mechanical ventilation, the administration of sedatives is often required for patient’s comfort and synchronization with ventilator. Thus, we tested the effect of common sedatives in intensive care unit (ICU) using our platform.

## Materials and Methods

### Cyclic stretch of lung epithelial cells

**Figure 1:**
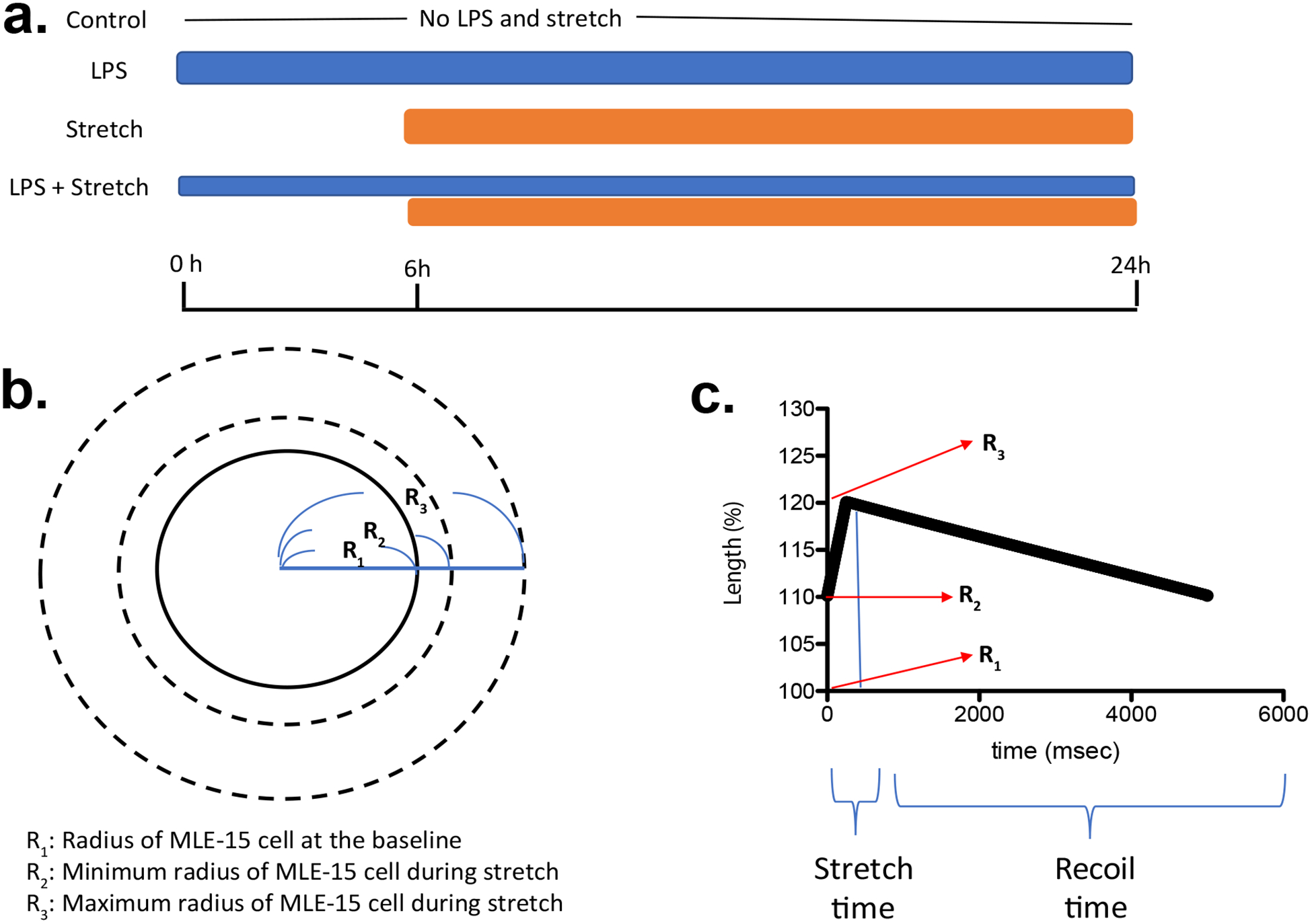
Scheme of in vitro cyclic stretch of MLE-15 cell (a) When MLE-15 cells are considered as a sphere, R_1_, R_2_ and R_3_ are defined as in the scheme. LPS, lipopolysaccharide. (b) Relationship between cell length and time is shown. Time for stretch will be written as S, and time for recoil will be written as R in the manuscript.

MLE-15 cells are immortalized mouse alveolar epithelial cells and kindly given by Dr. Rebecca Baron (Brigham and Women’s Hospital, MA, USA) [4]. MLE-15 cells were cultured in HITES medium containing L-gluta-mine, HEPES (10 mM), and 10% FBS. MLE-15 cells were plated as confluent monolayers on the BioFlex culture plates coated with collagen type I (Flexcell International, Hillsborough, NC) using the Flexcell® BioFlex® Cell Seeders. We subjected MLE-15 cells to the following treatments. 1) No treatment, 2) lipopolysaccharide (LPS, Sigma-Aldrich, St. Louis, MO, USA) (100 ng/mL) stimulation for 24 hours, 3) mechanical stretch initiated at 6-hour time point for 18 hours, 4) LPS stimulation at time point 0 hour, and mechanical stretch was added at 6-hour time point for 18 hours (Figure 1a). Usually mechanical ventilation is used for patients with pulmonary dysfunction, and group 4 mimicked this population. So far biaxial cyclic stretch has been used either in a sine wave [5–8], a square wave [5–9] or a triangular wave [5,10] to study VILI *in vitro*. In a sine wave, stretch time and relaxation time are equal. In a square wave, the cells reach to the maximum stretch right away. In a triangular wave, cells are stretched in a linear-ramped fashion, and a number of combinations can be chosen for stretch and relaxation time. In volume control and pressure control mechanical ventilation, lung undergoes gradual stretch with variable stretch and relaxation times, rather than abrupt stretch. Thus, biaxial mechanical stretch in a triangular wave was given to MLE-15 cells using Flexcell® FX-6000™ Tension System (Flexcell International). We assumed that cells at a basal state were round with a radius of R_1_ (Figure 1b). Minimum and maximum radius during stretch were defined as R_2_ and R_3_. TV would be calculated as 4π/3 × [(R_3_)^3^−(R_2_)^3^] under three-dimensional stretch, and we defined V = (R_3_)^3^−(R_2_)^3^. Tension to the cells at the radius of R_2_ would correspond to PEEP. Here we used the maximum elongation of 20% (R_3_ = 1.2 × R_1_). With the expectation that lung volume at the end of inspiration would be the same for both high and low TV ventilation strategy, we used the maximum elongation of 20% in this study. When R_2_ is 1.1 × R_1_ (10% elongation), V is equal to 0.429 (=V_1_). When R_2_ is 1.15 × R_1_ (15% elongation), V is equal to 0.239 (=V_2_). When R_2_ is 1.175 × R_1_ (17.5% elongation), V is equal to 0.138 (V_3_). As a result, V_1_/V_2_ and V_1_/V_3_ are 1.79 and 3.1, respectively. Because the ratio of high TV (12 mL/kg)/low TV (6 mL/kg) is 2, we considered elongation of less than 17.5% to correspond to low TV mimic stretch. Here we used 17.5% elongation as a model of low TV ventilation. We used 12 cycles/min for 18 hours using Flexcell® FX-6000™ Tension System (Figure 1a). The relationship between time and cell length (%) was shown in Figure 1c. At the end of experiments, we harvested cells and supernatant for analysis. In some of experiments, cells were co-incubated with sedatives propofol and dexmedetomidine at the doses indicated.

### Cell death analysis

We examined cell death using Annexin V apoptosis detection Kit (BD Biosciences, San Jose, CA, USA). Briefly, cells were harvested, and stained with both propidium iodide (PI) and Annexin V- FITC. Then, cells were subjected to flow cytometry using BD Accuri C6 (BD Biosciences). PI^−/low^ Annexin V^−/low^, PI^high^ Annexin V^−/low^, PI^−/low^ Annexin V^high^, and PI^−/low^ Annexin V^high^ cells were considered live, necrosis, early apoptosis and late apoptosis.

### Lactate dehydrogenase assay

Lactate dehydrogenase (LDH) is a stable enzyme released upon cell lysis and used as a surrogate of cell death. LDH level in the medium was measured per the company’s protocol (Promega, Madison, WI, USA).

### Cell surface expression on MLE-15 cells

We have measured Toll-like receptor (TLR) 2, TLR4, intercellular adhesion molecule-1 (ICAM-1) and receptor for advanced glycation endproducts (RAGE) on MLE-15 cells under different conditions using flow cytometry. TLR2, TLR4, ICAM-1 and RAGE were probed by anti-mouse TLR2 (CB225), TLR4 (SA15–21), ICAM-1 (YN1/1.7.4) (all from Biolegend, San Diego, CA, USA) and RAGE antibodies (697023) (R&D systems, Minneapolis, MN, USA), respectively.

### The nuclear factor k-chain-enhancer of activated B cells (NFkB) activation by supernatant of cultured MLE-15 cells

HEK-TLR2 cells (InvivoGen; San Diego, CA, USA) are HEK cells overexpressing TLR2 containing an NFkB-inducible secreted embryonic alkaline phosphate (SEAP) reporter [11]. They were either stimulated with TLR2 agonist Pam_3_CSK_4_ (100 ng/mL) (InvivoGen) or supernatant of cultured MLE-15 cells for 12 hours. NF-kB activation was assessed by using the reaction buffer Quanti-Blue (InvivoGen) to quantitate SEAP in the medium per the company protocol. Samples were subjected to a spectrophotometer analysis at 655 nm.

## Statistical Analysis

Data were statistically analyzed as indicated in the corresponding figure legends. Statistical analyses were performed with PRISM5 software (GraphPad Software, La Jolla, CA, USA). Statistical significance was defined as P < 0.05.

## Results

### Stretch speed was critical for cell death induction

**Figure 2:**
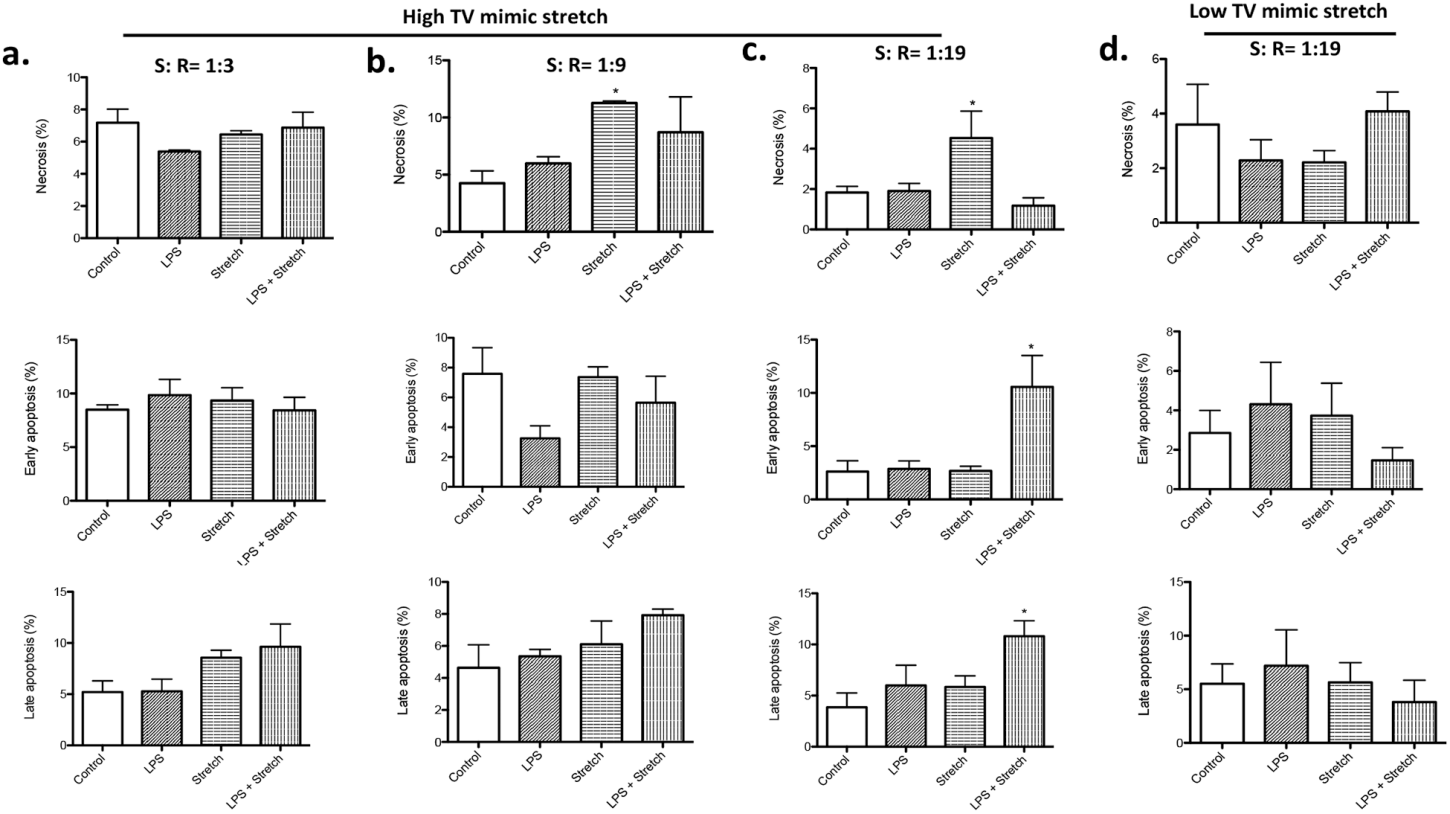
Cell death analysis of MLE-15 cells undergoing cyclic stretch mimic high and low TV ventilation Necrosis, early apoptosis and late apoptosis were determined in MLE-15 cells subjected to LPS stimulation, high TV mimic stretch and LPS + high TV mimic stretch at S: R= 1:3 (a), 1:9 (b) and 1:19 (c). MLE-15 cells were also subjected to LPS stimulation, low TV mimic stretch and LPS + low TV mimic stretch at S: R = 1:19 (d). The percentage of necrosis, early apoptosis and late apoptosis of all the MLE-15 cells was shown as mean +/− S.D. of triplicates. Statistical analysis was performed using one-way ANOVA with Bonferroni *post hoc* analysis. * denotes P< 0.05 versus control. TV, tidal volume; LPS, lipopolysaccharide.

First, we determined the optimal stretch pattern in a triangular wave to induce cell death in the high TV mimic stretch. We tested the three different stretch (S): recoil (R) ratio. While there was no difference in cell death under the four conditions at S: R = 1: 3 (Figure 2a), stretch slightly increased necrosis at S: R = 1: 9 (Figure 2b). However, LPS stimulation and stretch condition did not show any significant difference in cell death from the control. At S: R = 1: 19, stretch significantly induced necrosis, and the combination of LPS stimulation and stretch significantly increased the degree of apoptosis (Figure 2c). Because VILI in patients with pulmonary dysfunction undergoing mechanical ventilation is the usual clinical scenario, we considered the result of stretch study at S: R = 1:19 to represent this. We used this condition in all the subsequent experiments.

### High TV mimic stretch induced cell death, while low TV mimic stretch did not

**Figure 3:**
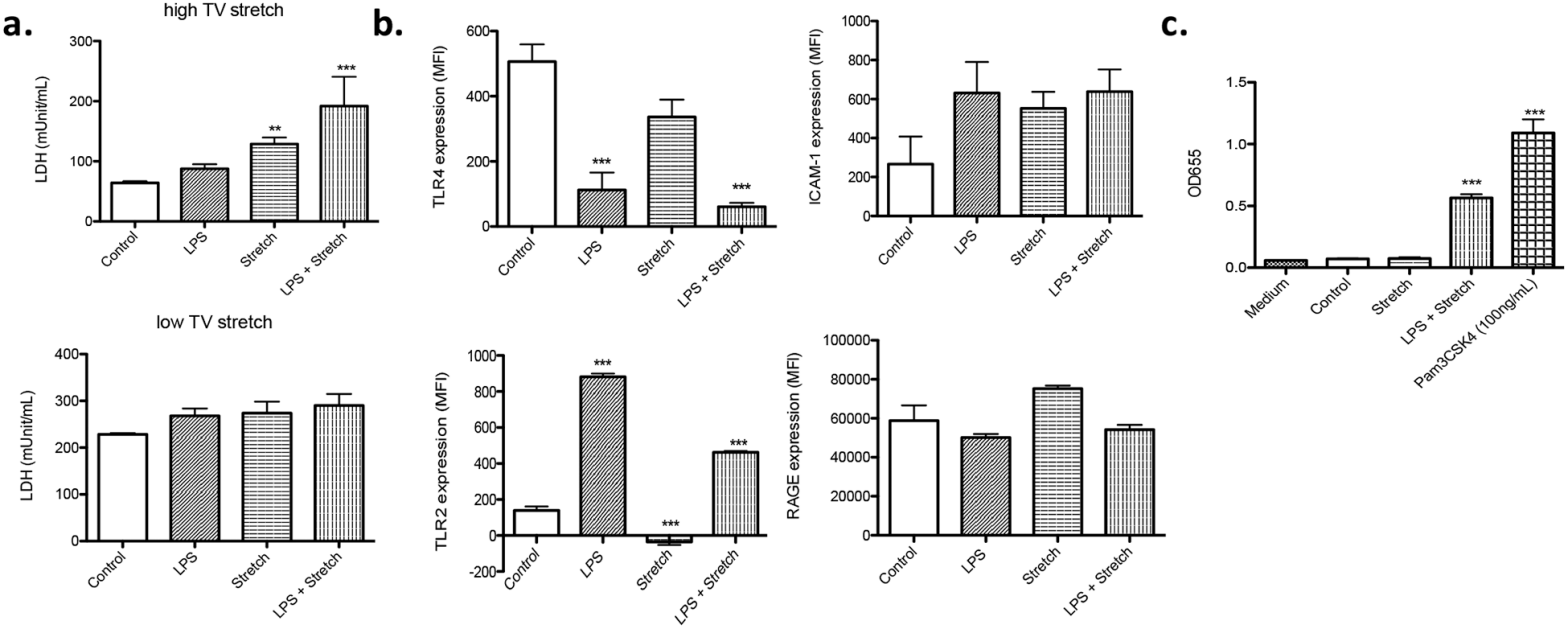
LDH release from MLE-15 cells and cell surface expression profiles (a) MLE-15 cells subjected to LPS stimulation, stretch and LPS + stretch at the S: R= 1:19. Stretch was done to mimic either high or low TV ventilation. LDH levels were measured in the supernatant of cultured MLE-15 cells. All the data were shown as mean +/− S.D. of triplicates. (b) Cell surface expression of TLR4, TLR2, ICAM-1 and RAGE was examined in MLE-15 cells under different conditions using flow cytometry. Data were shown as mean +/− S.D. of mean fluorescence intensity (MFI) of triplicates. (c) TLR2 activation was examined using HEK-TLR2 reporter assays as described in the Method. Statistical analysis was performed using one-way ANOVA with Bonferroni *post hoc* analysis. ** and *** denote P< 0.01 and P< 0.001 versus control, respectively. TV, tidal volume; LPS, lipopolysaccharide; LDH, lactate dehydrogenase; TLR, toll-like receptor; ICAM-1, intercellular adhesion molecule-1; RAGE, receptor for advanced glycation endproducts.

Stretch mimic low TV ventilation was tested at S: R = 1: 19. No difference of cell death was observed among all the groups (Figure 2d), which went along with the idea that this stretch was intended to mimic “lung protective” strategy. In both high and low TV mimic stretch, the maximum length of each cell was the same, indicating that the difference in the change of length by stretch was responsible for cell death. We also measured LDH level in the culture supernatant. In the stretch mimicking high TV ventilation, both stretch alone and the combination of LPS stimulation and stretch were associated with higher LDH levels in the medium (Figure 3a), compatible with the result of cell death analysis (Figure 2). In contrast, in the stretch mimicking low TV ventilation, there was no obvious difference in LDH levels among all the groups (Figure 3a).

### Stretch did not affect TLR2, TLR4, ICAM-1 and RAGE expression, while LPS stimulation affected TLRs expression

TLR4 is a receptor stimulated by LPS. Stretch mimicking high TV ventilation itself did not affect surface TLR4 expression, but LPS stimulation significantly reduced its cell surface expression (Figure 3b), suggesting that the signaling via TLR4 might have been downregulated over time after LPS stimulation. Lung epithelial cells express other TLRs such as TLR2 [12]. TLR2 can bind to a diverse array of ligands including Gram positive bacterial components, fungal components, and some of endogenous danger signals. Stretch did not affect TLR2 expression. However, LPS stimulation was associated with higher TLR2 expression (Figure 3b). TLR2 activation can induce apoptosis [13]. Endogenous danger signals such as heat shock proteins can bind to TLR2 [14]. We performed TLR2 activation reporter assay using the supernatant from cultured cells subjected to stretch studies mimicking high TV ventilation and found that TLR2 signaling pathway was activated in LPS-stretch condition (Figure 3c).

Because stretch could induce inflammatory reaction, we examined the expression of proinflammatory markers ICAM-1 and RAGE. We did not observe any difference in their expression under different conditions, indicating that inflammatory status might not be significantly different among the conditions tested (Figure 3b). These data indicated that our model would be good for understanding the mechanism of stretch related injury rather than proinflammatory cytokine mediated injury.

### The effect of sedatives on cell stretch induced cell death

**Figure 4:**
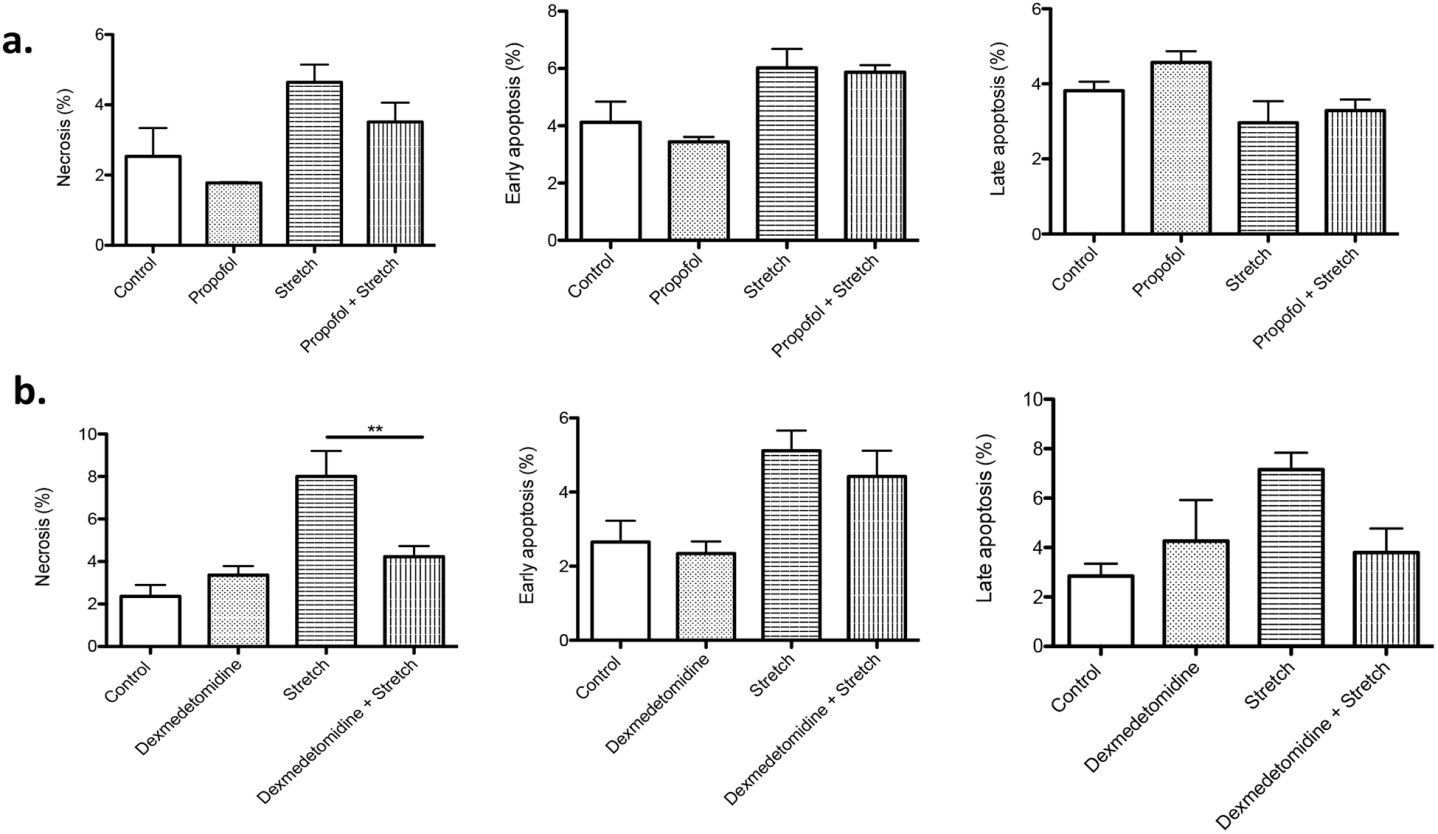
The effect of sedatives on cell death The effect of propofol (100 μM) (a) and dexmedetomidine (100 μM) (b) on high TV mimic cyclic stretch was examined. Necrosis, early apoptosis and late apoptosis were determined in MLE-15 cells subjected to stretch with or without sedatives at the S: R= 1:19. The percentage of necrosis, early apoptosis and late apoptosis of all the MLE-15 cells was shown as mean +/− S.D. of triplicates. Statistical analysis was performed using one-way ANOVA with Bonferroni *post hoc* analysis. ** denotes P< 0.01.

Sedation is an important part of treatment during mechanical ventilation so that patients can be comfortably ventilated. In ICU, intravenous drugs such as dexmedetomidine and propofol are often used. Thus, we tested the effect of these two sedatives on cell death induced by stretch. While propofol did not affect cell death, dexmedetomidine significantly attenuated the percentage of necrotic cells under stretch (Figure 4a and Figure 4b), suggesting that the selection of sedatives may be potentially important for mechanical ventilation management.

## Discussion

Here we described the cyclic stretch system mimicking high and low TV mechanical ventilation. While cyclic stretch mimicking high TV ventilation was associated with higher cell death, low TV mimetic did not affect cell death, in line with our clinical experience that low TV ventilation would be considered “lung protective”. Furthermore, dexmedetomidine attenuated the degree of necrosis under stretch mimicking high TV ventilation.

Volutrauma as a cause of VILI has been recognized for a long time. Low TV strategy has been used to attenuate volutrauma based on the landmark study by the ARDS Network [3]. However, use of low TV regimen does not completely abolish volutrauma. Injured lung is often heterogeneous, consisting of compliant (non/less-diseased) and non-compliant (diseased) parts. Even under low TV regimen, compliant part of the lung is likely subjected to larger stretch compared to non-compliant part, which can cause the former to be overstretched and succumb to volutrauma. Thus, it is important to understand the pathophysiology of VILI and develop therapeutic intervention. In contrast to injured lung, cells subjected to our stretch experiment are presumably homogeneous at the baseline. Thus, our high TV mimic cyclic stretch can mimic either injured lung ventilated with high TV or the compliant part of lung ventilated with low TV regimen. One notable thing is that the total length of stretch was not necessarily a determinant of cell death. Rather, the degree of change of cell length was associated with cell death, which further supports the role of low TV ventilation in lung protection.

While there are no therapeutics against VILI, the effect of sedatives/anesthetics on lung injury has been studied. In surgical suites, volatile anesthetics are often used and can be immumodulatory [15–22]. However, ventilation in surgical suites usually lasts for a very short duration and the lung in the majority of patients is not injured prior to ventilation. In contrast, in ICU, patients may need long term mechanical ventilatory assistance. Thus, it is clinically important to identify sedatives that potentially attenuate VILI. Although volatile anesthetics have been used as alternative sedatives in ICU in Canada and Europe [23], the administration of intravenous sedatives is typical practice in the majority of ICUs. Here, our data suggested that dexmedetomidine at the high dose might attenuate stretch-related cell death. The benefit of dexmedetomidine was shown in the preclinical models of VILI [24,25]. The involvement of α2 receptor on non-apoptotic cell death pathway has been described [26]. Whether or not the effect of dexmedetomidine on cell death in this stretch system is via α2 receptor will be examined in the future study. Despite the beneficial role of dexmedetomidine in VILI *in vitro* and in the preclinical models, it is unclear what the role of dexmedetomidine in VILI is in clinical scenarios. Further studies are needed to answer the question.

It is worth discussing the limitation of our study. Although we studied cell death in MLE-15 cells, lung consists of other cell types including alveolar macrophages. This can be potentially addressed by using primary lung tissue for stretching experiment. Macrophages/monocytes are major cytokine-producing cells. Our epithelial cell system is not necessarily a robust system to have proinflammatory responses as we observed. Thus, our system likely underestimates the component of biotrauma. Our system is devoid of vascular structure, which allows leukocytes to be recruited. When VILI develops, it is known that neutrophils are recruited to the site of injury to further worsen the lung injury. This phenomenon is currently difficult to recapitulate *in vitro*. Thus, *in vitro* stretch experiment needs to be complimentary with *in vivo* study.

In conclusion, we described the *in vitro* cyclic stretch system mimicking high and low TV ventilation. High TV mimetic was associated with increased cell death. Dexmedetomidine attenuated the degree of cell death in this system mimicking high TV ventilation, which needs further study in patients.
